# Child brain health in South Asia: moving from survival to prevention

**DOI:** 10.1016/j.lansea.2026.100760

**Published:** 2026-04-03

**Authors:** Jithangi Wanigasinghe, Nishani Lucas, Priyanka Madaan, Shahnaz Ibrahim

**Affiliations:** a Department of Paediatrics, University of Colombo and Lady Ridgeway Children's Hospital, Sri Lanka; b Department of Paediatrics, University of Colombo and De Soyza Maternal Hospital, Sri Lanka; c Pediatric Neurology Unit, Department of Pediatrics, Postgraduate Institute of Medical Education and Research, Chandigarh, India; d Department of Paediatrics & Child Health, Agha Khan University, Karachi, Pakistan

Child brain health does not begin at birth; it starts shaping much earlier—beginning before conception—and is influenced by the maternal–foetal–neonatal triad. A substantial proportion of early neurological outcomes are determined by events that unfold across this continuum.^1^ The first 1000 days; from conception to second birthday, are recognised as the most critical period for brain growth, neurodevelopment, and biological programming.^2^ However, current regional health priorities remain heavily weighted towards downstream outcomes such as neonatal survival, while upstream biological and social determinants of brain development are addressed insufficiently.

Maternal health inequities remain central to this discussion. Although the global maternal mortality ratio declined by about 40% between 2000 and 2023, progress remains too slow. In South Asia, poverty, nutritional insecurity, gender inequity, and educational disadvantage continue to shape maternal and child outcomes. Maternal anaemia, which affects 50% of adolescent girls and women in the region,^3^ is associated with prematurity, foetal growth restriction, and poorer developmental outcomes.

For South Asia to improve child neurological outcomes at population scale, health of women before and during pregnancy must become central. Despite major gains in maternal and child survival, survival to discharge for small and sick newborns is substantially lower.^4^ The main drivers of neonatal mortality—prematurity, congenital anomalies, hypoxic-ischaemic injury, infection, poor intrapartum care, and delayed access to specialist newborn care—are also major drivers of long-term neuro-disability among survivors. If the region is committed to reducing disability among surviving children, improving school readiness, and protecting future human capital, then investment must move towards prevention before injury occurs. Health of girls during adolescence deserves far greater attention in this agenda. It lays the biological and social foundation for healthy motherhood.

We must however note that South-Asia is not one story: regional success stories emphasize internal contrasts. World Bank Gender Data Portal reveals striking numbers within South-Asia.^5^ For example, Sri Lanka's female literacy is markedly higher (with gender parity) than the regional counterparts. Also, lower adolescent fertility in Sri Lanka (15 per 1000 girls aged 15–19) in 2023, than Bangladesh and Nepal (73 and 67 per 1000 respectively), contribute to improved maternal nutrition, health-seeking, birth preparedness, and infant care. Despite geography-related service delivery challenges, Maldives has high female literacy and accessible primary healthcare illustrating the role of social development in maternal-child outcomes. In contrast, larger countries such as India, Bangladesh and Pakistan carry a relatively higher burden due to population size and within-country inequities.

Several interventions deserve stronger and more explicit regional attention. Literacy, nutrition, and preparedness for pregnancy among women should be treated as child brain health priorities, not merely social welfare goals. This includes stronger enforcement against child marriage, underage pregnancy and protection of girls’ education. Adolescent health programmes should explicitly include nutrition, anaemia prevention, reproductive health education, and preparation for healthy pregnancy. Proven newborn interventions should be scaled up with quality assurance: neonatal resuscitation, essential newborn care, breastfeeding support, kangaroo mother care, infection prevention, and family-centred newborn care.^6^^,^^7^ Sustained immunisation coverage and stronger pro-immunisation attitudes are needed to reduce preventable neurological disease. Early developmental surveillance and timely intervention for at-risk infants should be expanded, as these can reduce disability and improve long-term function. Finally, paediatric neurologists must have a stronger voice in maternal, perinatal, and newborn policy discussions. Their contribution should not begin only after disability has appeared; it should help shape prevention at population level.

A stronger regional strategy with action on the maternal-foetal- neonatal triad is the need of the hour. South Asia has demonstrated that progress is possible, but a shift in direction is needed to drive our efforts to promote better outcomes. We propose a shift in policy and financing targets upstream to women and maternal health as a more effective strategy to secure child health across South Asia.

## Declaration of interests

All authors confirm no conflict of interest related to the content of the article.
